# Simple Microcontact Printing Technique to Obtain Cell Patterns by Lithography Using Grayscale, Photopolymer Flexographic Mold, and PDMS

**DOI:** 10.3390/biomimetics7040155

**Published:** 2022-10-08

**Authors:** Rocio Gimenez, Camilo Pérez-Sosa, Natalia Bourguignon, Santiago Miriuka, Shekhar Bhansali, Carlos R. Arroyo, Alexis Debut, Betiana Lerner, Maximiliano S. Pérez

**Affiliations:** 1 IREN Center, National Technological University, Buenos Aires 1706, Argentina; 2 Department of Electrical and Computer Engineering, Florida International University, Miami, FL 33174, USA; 3 LIAN-CONICET-FLENI, Ruta 9 Km 52, 5, Belén de Escobar 1625, Argentina; 4 Centro de Nanociencia y Nanotecnología, Universidad de las Fuerzas Armadas ESPE, Sangolqui P.O. Box 171-5-231B, Ecuador; 5 Collaborative Research Institute Intelligent Oncology (CRIION), Hermann-Herder-Straße 4, 79104 Freiburg im Breisgau, Germany

**Keywords:** grayscale, PDMS stamp, flexographic photopolymer mold, contact printing, 3D patterns

## Abstract

Microcontact printing using PDMS embossing tools and its variations have aroused the interest of a wide spectrum of research fields, hence the feasibility of defining micro and nanoscale patterns. In this work, we have proposed and demonstrated a novel lithography method based on grayscale patterns printed in a flexographic photopolymer mold and transferred to epoxy resin and a single PDMS stamp to obtain different microprint pattern structures. The geometry of the patterns can be modified by adjusting the layout and grayscale of the stamp patterns. The functionality of this contact printing methodology was validated by generating human induced pluripotent stem cells (hiPSC) patterns. These specific micropatterns can be very useful for achieving complex differentiation in cell lines such as hiPSC. Microfabrication through the new technique provides a promising alternative to conventional lithography for constructing complex aligned surfaces; these structures could be used as components of biological patterns or microfluidic devices.

## 1. Introduction

Surface patterning has become an intensively researched area within material science and engineering [1]. A controlled topography can influence the optical [2], wetting [3,4], or adhesion properties [5,6] of a surface. Moreover, it has been exploited for a wide variety of applications that include superhydrophobic surfaces [7], smart windows [8], electronics [9], and biomedical purposes [10], among others. A wide range of technologies can be used to create these types of patterns, such as photolithography, surface wrinkling, soft lithography, microinjection molding, casting, and micro hot embossing. 

Furthermore, an even more complex step is the creation of these patterns in three dimensions (3D), obtaining elements with different heights or shapes [11]. This type of complex micro and nanostructure can be achieved with technologies for modeling three-dimensional (3D) surfaces; grayscale lithography is one of the most known [12]. In grayscale lithography, the dose of ultraviolet light (UV) exposure is spatially modulated to locally control the crosslinking or dissolution rate of a material, generally a polymer or resin sensitive to ultraviolet (UV) rays. This material is used with grayscale masks [13,14,15] or direct writing systems [16,17,18] to obtain structures with precise and arbitrary lateral and vertical dimensions. 

Direct writing approaches typically involve expensive equipment that “writes” the design pixel by pixel, leading to low production speeds. These include Focus Ion Beam [19], Electron Beam [20], and Laser Lithography [21]. On the other hand, techniques that use physical gray tone masks can generally expose larger areas simultaneously, thus enabling mass production. However, the cost and time required to manufacture these masks hinder rapid prototyping, especially considering that a different mask is required for each pattern and that increasing the pattern’s complexity also increases the mask’s complexity. The main method for this approach is grayscale photolithography [14,22,23], while other lesser-known methods are based on X-ray [24] or holographic lithography [25].

Once these patterns are obtained on a surface, they can further function as molds or stamps to replicate the same design with other materials. A particularly suitable technique for this is microcontact printing (µCP), which uses the relief pattern on the surface of a stamp to form patterns on the surfaces of substrates [26]. The stamp is ‘inked’ with a solution or material and brought into conformal contact with the surface of the substrate, transferring the ‘inked’ material only in the regions where the stamp makes contact with the substrate. This technique has been used to obtain arrays of nanoparticles [27], proteins [28], bacteria [29], and even DNA [30], thus, finding applications in biosensors, diagnostic immunoassays, chromatography, DNA microarrays, cell culturing, and further analytical procedures.

Previously, we reported and patented a simple and accessible process for the fabrication of polydimethylsiloxane (PDMS) microdevices by using a mold from a printing plate photopolymer called Flexcel as a master mold (Fmold) [31,32]. The Flexcel photopolymer enabled the transfer of these patterns to PDMS replicas with high reliability. The use of the Fmold allows the fabrication of master molds with dimensions up to 1270 × 2062 mm^2^ and structure heights ranging from 53 to 1500 μm [31]. This flexographic method presents advantages such as (a) low cost, (b) availability, (c) non-cleanroom facilities, (d) short time mold fabrication, (e) high durability of molds, (f) low surface roughness of the structures, and (g) the possibility of high-throughput production of epoxy resin molds and PDMS replicas with high precise replication [31,33]. Moreover, another previous work already reported the use of Fmold to create multilevel molds and then transfer the structures to PDMS replicas [34]. There, the different heights archived on the PDMS mold were controlled by modifying the UVA exposure time, so when the exposure time was longer, the height of the structure decreased, and vice versa. Herein, the novelty of the work is the manufacturing method based on photopolymers to create complex 3D patterns with a simple/basic grayscale mask using a Flexcel flexographic photopolymer. Unlike previously reported methods [31,33,34], the exposure time remained the same throughout the plate and the different heights present within the mold were archived by means of the grayscale mask. We show that a proper grayscale mask design is critical to fabricating pattern structures. The technology was validated using the pattern created on the PDMS surface as a template for microcontact printing in order to generate a respective pattern of human induced pluripotent stem cells (hiPSC) on the cell culture plates treated with reagents for adhesion.

## 2. Materials and Methods

### 2.1. Fabrication of PDMS Stamp

**Figure 1:**
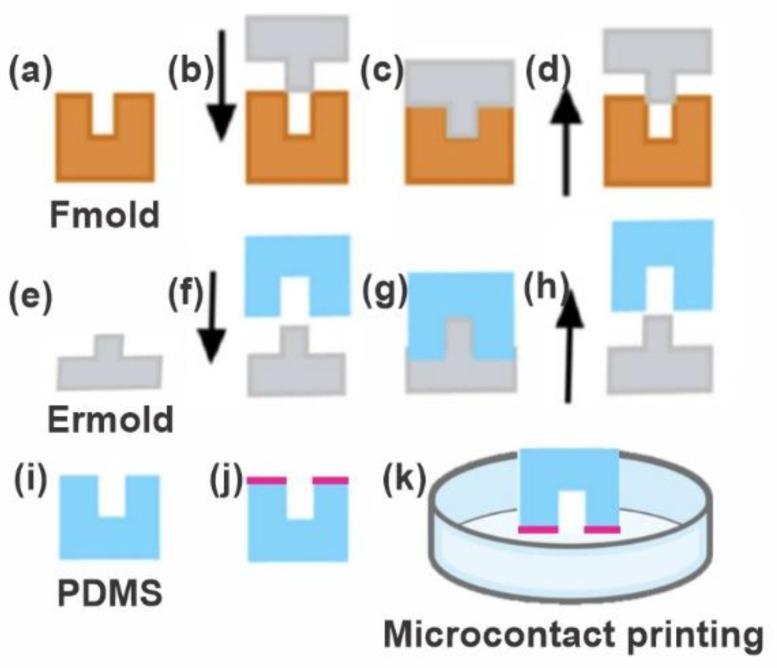
PDMS stamp fabrication. (**a**) Photopolymer flexographic master mold (Fmold). (**b**,**c**) The epoxy resin mold (ERmold) is formed on the Fmold and cured at room temperature. (**d**,**e**) The ERmold is demolded after 72 h. (**f**,**g**) The PDMS is cast on the ERmold and cured at 40 °C overnight. (**h**,**i**) The PDMS replica is peeled off. (**j**,**k**) The PDMS stamp is used for microcontact printing.

Figure 1 schematizes the manufacture of a PDMS stamp using a flexographic photopolymer-based manufacturing method that shows the main steps involved in the production of the photopolymer mold, the epoxy resin mold, and the PDMS replica.

#### 2.1.1. Photopolymer Flexographic Mold (Fmold)

The photopolymer Flexcel NX and Thermal Imaging Layer (TIL) in the fabrication of the molds were supplied by Eastman Kodak [35]. The photopolymer thickness was 1.14 mm. The grayscale mask was designed with Layout editor 20220423 software [36] using the amplitude modulated (AM) approach, a conventional screening technique that modifies the size of the dot but maintains the space between the dots fixed. The design was subsequently transferred to the TIL using an infrared laser source (2400 ppi), and then it was laminated onto the unexposed photopolymer plate. Following this step, the photopolymer plate was exposed to UVA light at 0.45 J on the reverse side, and then the front side was exposed to UVA light at 19 J for 360 s. The exposure to UVA wavelengths crosslinks the organic compounds on the photopolymer plate. Subsequently, the TIL was removed. After UVA exposure, the photopolymer plate was washed with the solvent PROSOL N-1 (Eastman Kodak) at 360 mm/min and dried in an oven at 50 °C for 30 min. Lastly, the photopolymer plate was exposed to UVC light at 10 J for 17 min and UVA light on the front side at 4 J for 2 min. The UVC wavelengths are used to end the reaction, thus obtaining large, stable, and insoluble molecules. The photopolymer mold accordingly obtained was denominated Fmold [31,37].

#### 2.1.2. Epoxy Resin Mold (ERmold)

Before creating the corresponding epoxy resin mold, the Fmold was placed in an oven for 12 h at 100 °C and then in a vacuum chamber for 1 h at room temperature. A cleaning process was then performed with 70% ethanol solution for 7 min in an ultrasonic bath, and later the Fmold was dried in an oven for 10 min at 40 °C. Finally, the Fmold was cleaned with a nitrogen stream. The epoxy resin and curing agent (Cristal-Tack, Novarchem-Argentina, Villa Martelli, Argentina) were mixed in a 2:1 wt ratio and gently stirred for 3 min. The mixture was sonicated (TESTLAB Ultrasonic Cleaner, Warsaw, Poland) to remove the air bubbles for 7 min. The epoxy resin was then slowly poured over the Fmold and cured for 72 h at room temperature. Finally, the epoxy resin mold (ERmold) was peeled off from the Fmold [33].

#### 2.1.3. PDMS Stamp

Polydimethylsiloxane (PDMS) was mixed in a 10:1 wt ratio with a curing agent (Sylgard 184 Silicone Elastomer Kit, Germantown, WI, USA), as previously described in Peñaherrera et al. [38]. Subsequently, to remove air bubbles, the mixture was placed in a vacuum chamber for 30 min. Then, it was poured over the ERmold and cured overnight in an oven at 40 °C. Next, the PDMS stamp was peeled off from the ERmold, and inlet and outlet holes were punched with a 1 mm diameter biopsy puncher (Integra Miltex^®^Ted Pella, Inc., Davies Drive, CA, USA). 

### 2.2. Characterization

Before characterization, the PDMS stamp was blown with nitrogen gas to remove dust, followed by an ultrasonic bath in ethanol (70% *v*/*v*) for 10 min (repeated 5 times) and finally dried in an oven for 1 h at 40 °C. Morphological characterization was carried out using a Field Emission Gun Scanning Electron Microscope (TESCAN FEG SEM MIRA3, Brno, Czech Republic). The molds were previously metalized with a 20 nm gold layer, and SEM micrographs were taken at 5 kV to avoid damaging the samples. In addition, profilometry measurements were carried out with a Dektak XT profilometer from Bruker (Billerica, MA, USA). Linear scans were conducted at a scan speed of 22.75 µm/s with a 25 µm radius tip and a sampling rate of 0.01 Hz/mm. The analysis was performed with Vision 64 software. 

### 2.3. Cell Culture

Human induced pluripotent stem cells (hiPSC) were used. This cell line was modified to express a fluorescent protein (H2B-Celurean), by means of the piggyBac method. The cell line was maintained in an E8 Flex medium (GIBCO), supplemented with Rock Inhibitor, in adherent cell culture plates functionalized with 1× Geltrex ^®^(GIBCO, Waltham, MA, USA).

### 2.4. Contact Printing on Cell Culture Plates

To obtain the impression of the PDMS seal on the adherent culture plate, a chemical transfer methodology was used, mixing PEG (Polyethylene glycol 6000 SIGMA), PEGDA (Poly (ethylene glycol diacrylate) and a photoinitiator ((2-Hydroxy-4′-(2-hydroxyethoxy)-2-methylpropiophenone). For crosslinking on the surface, the three components were mixed until they formed a homogeneous substance. After this, the mixture was placed in a volume that completely covered the multiwell plate. To functionalize the crosslinking, the UV oven (UVP UltraViolet Crosslinker Model Cl-1000, Upland, CA, USA) was used for 8 min at maximum intensity until evidencing the adherence of the mixture on the surface and the absence of lumps. After this, the seal was left overnight on the surface until the impression was completely transferred. Hoang, et al. [39], reported the concentrations and methodology in more detail used in this work.

## 3. Results and Discussion

**Figure 2:**
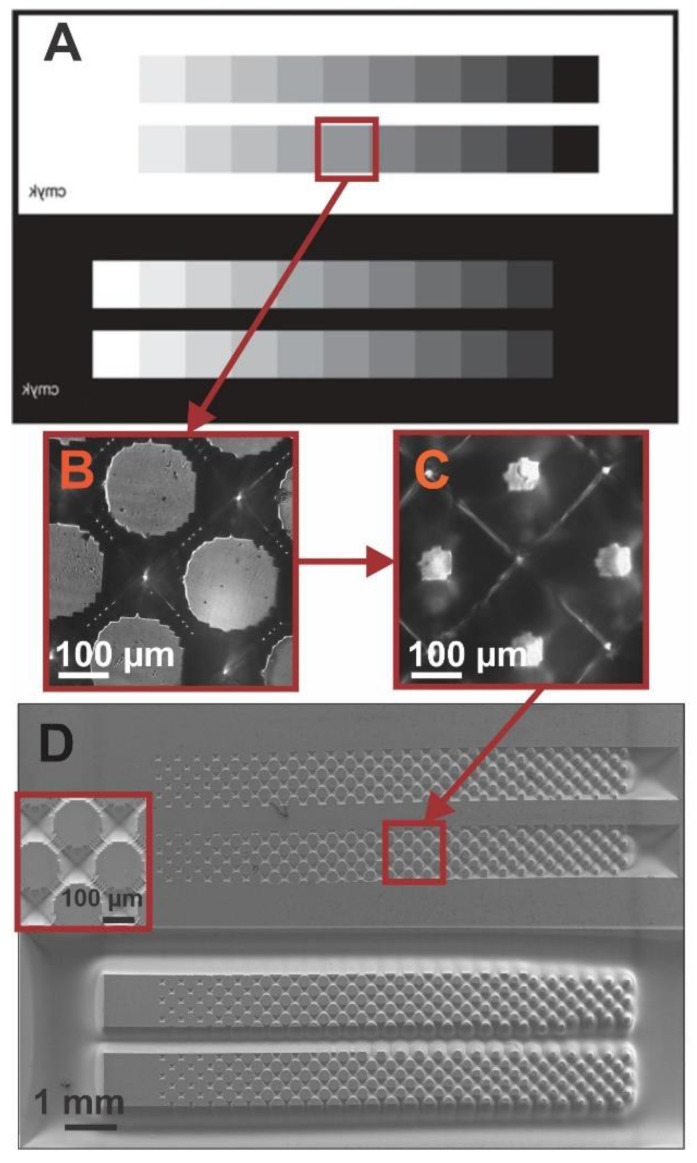
(**A**) Design of the grayscale mask used. (**B**) Optical image of the resulting pattern in the photopolymer flexographic mold (Fmold). (**C**) Optical image of the resulting pattern in the epoxy resin mold (ER mold). (**D**) SEM image of the PDMS stamp. The inlet shows a magnification of the respective pattern obtained with the mask.

The method of fabrication based on flexographic photopolymer molds used in this work [33] requires the use of a mask. Therefore, we designed and used a simple grayscale mask where one end is entirely opaque (blocks UV light) and gradually becomes more transparent (lets UV light pass through) (Figure 2A). To create this grayscale mask, we employed an array of dots of different sizes but with a fixed spacing, where the largest dots were located in the opaquer part of the mask, and vice versa.

As seen in Figure 2, we start from a relatively simple mask containing only squares of different gray tonalities (Figure 2A) to obtain a complex pattern of 3D structures (Figure 2D). Using the grayscale mask, we first obtain a pattern of dots in the photopolymer flexographic mold (Fmold) (Figure 2B) and a complementing structure in the epoxy resin mold (ER mold) (Figure 2C). Figure 2D shows the final PDMS stamps obtained with the 3D pattern of the design on their surface. The relief seen on PDMS replicas gradually varies in pattern and depth when viewed from one end of the stamp to the other (Figure 2D), replicating the pattern obtained in the Fmold. 

**Figure 3:**
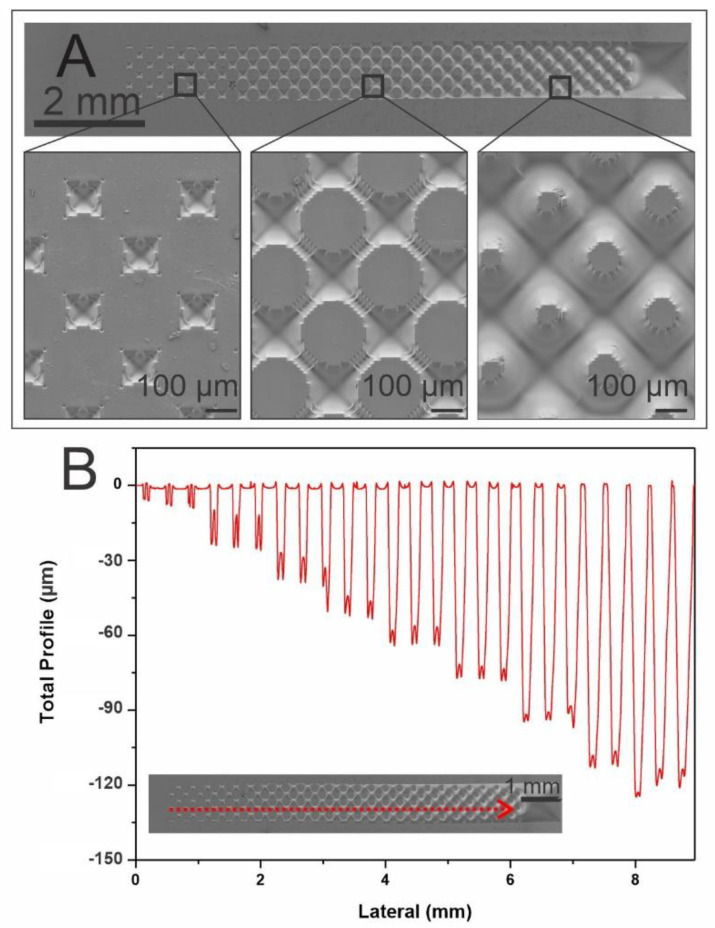
(**A**) SEM images of the 3D patterns of the PDMS stamp obtained from ERmold showing a magnification in three different areas. (**B**) profilometry profiles of the 3D structures of the PDMS replica obtained. Insert: SEM image shows the path on the structure profiled.

Figure 3A shows the SEM characterization of the forming patterns of circles obtained in the PDMS stamp which vary from smaller to larger diameters when going from a darker to a lighter grayscale, while the height is the same for all structures. Figure 3B shows profilometry profiles of the obtained 3D structures, where the depths of the cavities surrounding the circle patterns change from 10 µm to 150 µm. As seen in Figure 3A, on the right side of the stamp, we observe an array of dots, with diameters of approximately 100 µm, that are surrounded by cavities up to 130 µm deep. As one moves to the left side of the stamp, the dots begin to increase their size, gradually decreasing the space between them until finally, on the left side of the stamp, they interconnect, forming a network of dots. The depth of the cavities surrounding them also gradually diminishes until reaching a depth of 10 µm (Figure 3B). It should be noted that in the top image of Figure 2D and of Figure 3A we observe two pyramidal depressions that correspond to the black square of the grayscale mask. These depressions are observed instead of a pattern because the dots that should be formed are too small for the resolution of the technique, thus directly creating the depression. In addition, because the walls that are formed in this process are angled and not straight, the structure created has a pyramidal shape.

Our previous work already reported a flexographic photopolymer manufacturing method to create multilevel molds [34]. There, the different heights archived on the PDMS mold were controlled by modifying the UVA exposure time, so when the exposure time was longer, the height of the structure decreased, and vice versa. However, in the present work, the exposure time remained the same throughout the plate and the different heights present within the mold were archived by means of the grayscale mask.

Using a grayscale mask with the conventional methods, such as photolithography and deep reactive ion etching, can generally obtain only basic reliefs such as ramps, which may even have limited resolution [22,40]. To achieve the same pattern that we obtained in this work using the traditional methods, the mask would have to be incredibly complex [41]. However, we were able to show that we can obtain this complex pattern using this simple mask, given the grayscale nature of the mask with dots of different sizes at a constant distance, in conjunction with the flexographic photopolymer fabrication method. Another difference observed with the photopolymer method is that the pattern obtained has the same height and cavities around it, whereas with the other methods, the reverse is typically obtained (i.e., the pattern has different heights). Moreover, by creating the PDMS mold from the ERmold that is a copy of the Fmold [31,33], we can obtain this type of inverse geometry. 

Figure 3A shows the SEM characterization of the forming patterns of circles obtained in the PDMS stampwhich vary from smaller to larger diameters when going from a darker to a lighter grayscale, while the height is the same for all structures. Figure 3B shows profilometry profiles of the obtained 3D structures of the PDMS replica, while the depths of the circle patterns change from 10 µm to 150 µm.

One of the main disadvantages of using an elastomeric stamp that exhibits high compressibility (Young’s modulus ≈ 3 MPa [42]) is that it can collapse as a result of buckling or sticking laterally, for example [43]. This is especially true for stamps with high aspect ratios and can lead to defects on the pattern such as deviations in size and shape, or even printing in areas that must remain ink-free. However, a feature that can help avoid this type of drawback is that the stamp obtained in this work presents trapezoidal shapes, especially perceptible in areas with high aspect ratios. In this manner, stamps manufactured with flexographic photopolymer, either using grayscale or conventional designs, would be suitable as stamps in structures with a high aspect ratio. It is also important to remark that by using this technique, we can generate these patterns in a single exposure in a large area (1270 × 2062 mm^2^) as we reported for the Flexcel technique [31].

This embossed PDMS has subsequently used a stamp for microcontact printing to transfer molecules of interest with the desired pattern onto a surface. To generate cell patterns of human induced pluripotent stem cells (hiPSC), the cell culture plates were functionalized by mixing PEG, PEGDA and photo initiator. For this, the reagents were mixed until a homogeneous mixture was obtained and the surface was activated by UV light. After that, the PDMS stamp with the 3D patterns obtained was placed on the surface for 6 to 8 hours. Once the process was finished, the PDMS stamp was removed from the functionalized surface, leaving the printed pattern. For the seeding of the hiPSCs, 1× Geltrex was added on the previously treated surface. The geltrex was left for 1 h and 10,000 cells per ml were seeded.

**Figure 4:**
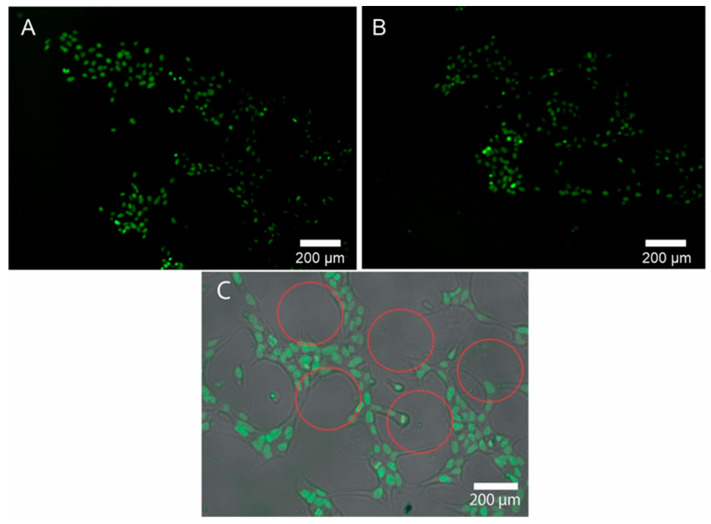
(**A**) Adhered hiPSC cells in the pattern printed on the culture plate. (**B**) Cells attached to a pattern of different heights with higher cell densities after 12 h of incubation. (**C**) Brightfield and overlay layer that show how the cells follow only the segment where the pattern was printed. Red outlines show the dots of the patterns created by the microcontact printing technique; the cells grew around the dots.

Figure 4A shows how the cells adhered to the parts of the pattern where the Geltrex could functionalize. The black circles without cells correspond to the design of the PDMS stamp. In Figure 4B, it can be seen how the cells continued in time, forming cell aggregates with a greater number of cells but maintaining the segment of the pattern previously printed by the PDMS stamp.

Microcontact printing (µCP) can be very useful in the biological field to create patterns that allow achieving complex differentiation in cell lines such as hiPSC. It is known in advance that the surface is mainly responsible for biological performance [44]. The extracellular matrix is directly responsible for the signals emitted by cells which regulate cell behavior and fate [45]. Adding to micropattern technology, µCP tools generate promising alternatives to improve the biological performance of many experiments associated with cell culture and differentiation events, gene expression, and extracellular communication, among others [44]. In the present work, we demonstrate a simple and inexpensive system to be able to generate a µCP that produces patterns for cell cultures in the hiPSC line.

## 4. Conclusions

We successfully demonstrated the fabrication of a complex 3D pattern on the surface of PDMS molds using a single step with a grayscale mask and flexographic photopolymer mold. The Fmold method, in conjunction with a grayscale mask, allowed us to create this elaborate pattern in a cost- and time-effective manner. We then proved that the embossed mold could be effectively employed as a stamp using microcontact printing in order to replicate that pattern on cell culture plates with induced human induced pluripotent stem cells. This up-and-coming tool will facilitate differential differentiation in stem cells based on pattern changes on the growth surface.

This grayscale mask and Fmold lithographic approach can be used to create a variety of three-dimensional topographies and structures in a single high-resolution lithographic step while enabling high-throughput, larger area fabrication of structures than ever before reported, with rapid reconfiguration. The versatility of this technique allows for the rapid prototyping of micromolds and relief structures that can be incorporated into devices with chemical and biological applications, microfluidics, and microelectromechanical systems.

## Data Availability

Not applicable.
